# Rigidly foldable origami gadgets and tessellations

**DOI:** 10.1098/rsos.150067

**Published:** 2015-09-16

**Authors:** Thomas A. Evans, Robert J. Lang, Spencer P. Magleby, Larry L. Howell

**Affiliations:** 1 Department of Mechanical Engineering, Brigham Young University, Provo, UT 84602, USA; 2 Lang Origami, Alamo, CA 94507, USA

**Keywords:** origami, rigid foldability, tessellation, crease pattern

## Abstract

Rigidly foldable origami allows for motion where all deflection occurs at the crease lines and facilitates the application of origami in materials other than paper. In this paper, we use a recently discovered method for determining rigid foldability to identify existing flat-foldable rigidly foldable tessellations, which are also categorized. We introduce rigidly foldable origami *gadgets* which may be used to modify existing tessellations or to create new tessellations. Several modified and new rigidly foldable tessellations are presented.

## 1. Introduction

An important characteristic of origami structures is rigid foldability. An origami tessellation is rigidly foldable if all sectors remain rigid and deflection only occurs at the crease lines. Because many materials used in engineering are much stiffer than paper, non-rigidly foldable tessellations (those that require deflection of the paper sectors) may have restricted movement when constructed out of these rigid materials. However, rigidly foldable tessellations may be constructed using stiff materials, leading to potential applications such as architecture and deployable arrays.

The study of origami has inspired engineering applications in recent years, including deployable structures such as solar panels [1,2] and sterile shrouds [3], as well as sandwich panel cores [4], self-folding membranes [5], a self-deployable origami stent graft [6] and tunable metamaterials [7]. Origami techniques have also been used in packaging [8] and an origami-folding robot has been developed [9]. Origami is also of interest to the architectural community and has been used as inspiration in the design of a timber constructed chapel in Switzerland [10].

Origami research has focused on several related areas. Hull developed theorems which govern flat-foldable origami patterns [11] and evaluated the possible crease assignments which result in flat-foldable origami [12]. Schenk & Guest [13] described the kinematics of twofolded metamaterials based on the Miura-ori fold pattern. Wei *et al.* [14] characterized the elastic response of a simple periodically folded Miura-ori structure. Demaine *et al.* [15] showed that adding additional creases to an origami model can allow the model to be mathematically folded.

Research on rigidly foldable origami has addressed the issue in several different ways. Huffman derived basic relationships between the various dihedral angles in a rigidly foldable degree-4 polyhedral vertex using spherical trigonometry [16]. Wu used quaternions and dual quaternions to model rigid origami [17], resulting in a system of six nonlinear equations where constrained nonlinear optimization was used to converge to a solution. Kinematic analysis of origami, where the creases are modelled as hinges and the panels as rigid links, has provided insight to rigid foldability of crease patterns [18–20]. Similar approaches have also been employed to evaluate the stability of rigid foldable tessellations [21,22]. Tachi developed conditions for partially folded quadrilateral surfaces [23]. These methods resulted in systems of nonlinear equations, requiring optimization techniques to converge to solutions. Tachi also developed equations which compare the dihedral angles of quadrilateral mesh origami [24].

A method for analysing the rigid foldability of origami patterns composed entirely from flat-foldable, degree-4 vertices has been developed previously by the authors [25]. While mathematically equivalent to Tachi's matrix formalism [23,24], it has a simple geometric interpretation that facilitates evaluation of rigid foldability, in many cases, by inspection alone. In this paper, we use this method to evaluate previously existing tessellations for rigid foldability. These tessellations are characterized and a comparison of these tessellations is presented. We develop several new origami *gadgets*, which are tools in the modification and creation of rigidly foldable tessellations. These gadgets are used in two different ways. First, they are used to replace portions of existing rigidly foldable tessellations to create slightly modified tessellations. Second, the gadgets are tessellated to make new rigidly foldable patterns.

## 2. Rigidly foldable origami

An origami pattern is said to be rigidly foldable if all panels remain rigid while all deflection occurs at the crease lines during deployment. We previously presented a method for determining if an origami pattern composed of degree-4 vertices is rigidly foldable [25]. Because of its relevance to this work, the method is briefly reviewed here.

**Figure 1.**
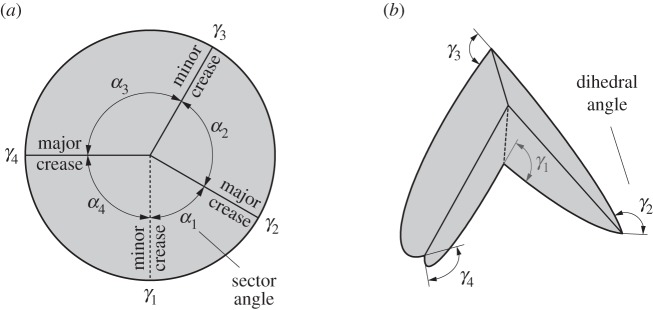
A degree-4 origami vertex in its (*a*) unfolded and (*b*) partially folded states. Mountain folds are indicated by solid lines and valley folds by dashed lines.

In this paper, we focus on patterns composed of degree-4 vertices. As illustrated in figure 1, a degree-4 vertex is formed by the junction of four creases. The paper between adjacent creases is called a *sector* and the angle between adjacent creases is called a *sector angle*, designated *α*. The sum of all sector angles around a vertex is 2*π*. The angle of the fold itself is the *dihedral angle* or *fold angle*, denoted by *γ*, which is the angle between the surface normals of the two incident sectors. A crease may be a *valley fold* (*γ*>0), a *mountain fold* (*γ*<0) or *unfolded* (*γ*=0). We will indicate mountain folds by solid lines and valley folds by dashed lines. We index the sector angles *α*_*i*_ and dihedral angles *γ*_*i*_ so that sector *α*_*i*_ lies between folds *γ*_*i*_ and *γ*_*i*+1_, as illustrated in figure 1.

### 2.1 Flat-foldability in degree-4 vertices

A flat-foldable vertex begins in an initial flat position and can be folded to achieve a secondary flat position. All dihedral angles are equal to ±*π* in the secondary flat position of a degree-4 vertex. Likewise, an origami pattern composed entirely of degree-4 vertices is considered flat foldable if there exists a configuration where all dihedral angles in the pattern are equal to ±*π*. The conditions for flat-foldability are well known (e.g. [12]); for degree-4 vertices, they are as follows:

— there exist threefolds of one parity and onefold of the other;
— the smallest angled sector, if unique, is incident to folds of opposite parity (‘anto’);
— the largest angled sector, if unique, is incident to folds of the same parity (‘iso’); and
— opposite sector angles sum to *π*;

where a fold's ‘parity’ refers to its assignment as a mountain or a valley fold.

If there exist two equally valued smallest angled sectors, at least one of these sectors must be anto and its opposite sector must be iso. We call the two opposite creases with opposite parity the *minor* creases and the two opposite creases with equal parity the *major* creases.

These conditions give us the following relationship between sector angles in a degree-4 vertex [11]:

2.1 $$\alpha_{1}+\alpha_{3}=\alpha_{2}+\alpha_{4}=\pi .$$

These are necessary conditions, not sufficient; an origami pattern composed entirely from flat-foldable vertices may self-intersect before the final flat state. Such a pattern is considered non-flat-foldable. Also, any origami tessellation containing one or more non-flat-foldable vertices cannot be flat-foldable.

### 2.2 Fold-angle multipliers

We will use fold-angle multipliers to evaluate tessellations. Fold-angle multipliers, as defined in [25], define the ratio between the half-angle tangent of adjacent dihedral angles in an origami vertex. This ratio is constant in any flat-foldable degree-4 origami vertex throughout the entire motion of the vertex [23].

The following relationships between dihedral angles in a flat-foldable degree-4 origami vertex are given in [25]:

2.2 $$\gamma_{3}=-\gamma_{1}$$

and

2.3 $$\gamma_{2}=\gamma_{4}=2\arctan\left(\frac{\sin((1/2)(\alpha_{1}+\alpha_{2}))}{\sin((1/2)(\alpha_{1}-\alpha_{2}))}\tan\left(\frac{1}{2}\gamma_{1}\right)\right).$$

The fold angle multiplier, *μ*_*i*_, for the *i*th sector is defined as follows:

2.4 $$\mu_{i}\equiv \frac{\tan((1/2)\gamma_{i+1})}{\tan((1/2)\gamma_{i})}.$$

Using equations (2.2)–(2.4), the multipliers for each sector in a vertex may be evaluated as follows:

2.5 $$\begin{array}{rl} \mu_{1} & \equiv \frac{\tan((1/2)\gamma_{2})}{\tan((1/2)\gamma_{1})}=\frac{\sin((1/2)(\alpha_{1}+\alpha_{2}))}{\sin((1/2)(\alpha_{1}-\alpha_{2}))}, \end{array}$$

2.6 $$\begin{array}{rl} \mu_{2} & \equiv \frac{\tan((1/2)\gamma_{3})}{\tan(1/2\gamma_{2})}=-\frac{1}{\mu_{1}}=-\frac{\cos((1/2)(\alpha_{2}+\alpha_{3}))}{\cos((1/2)(\alpha_{2}-\alpha_{3}))}, \end{array}$$

2.7 $$\begin{array}{rl} \mu_{3} & \equiv \frac{\tan((1/2)\gamma_{4})}{\tan((1/2)\gamma_{3})}=-\mu_{1}=\frac{\sin((1/2)(\alpha_{3}+\alpha_{4}))}{\sin((1/2)(\alpha_{3}-\alpha_{4}))} \end{array}$$

2.8 $$\begin{array}{rl} \;\text{and}\;\;\mu_{4} & \equiv \frac{\tan((1/2)\gamma_{1})}{\tan((1/2)\gamma_{4})}=\frac{1}{\mu_{1}}=-\frac{\cos((1/2)(\alpha_{4}+\alpha_{1}))}{\cos((1/2)(\alpha_{4}-\alpha_{1}))}. \end{array}$$

There is a special case to note: when the major crease fold lines are collinear (*α*_2_+*α*_3_=*π*), zero and infinite fold-angle multipliers are obtained. This occurs because the major crease lines must be completely folded before the minor crease lines begin folding. Because such vertices have more than 1 d.f., they are not considered in this paper.

### 2.3 Rigidly foldable polygons

A polygon in a flat-foldable origami tessellation is rigidly foldable only if the product of all fold-angle multipliers in the polygon is equal to one [25]. Therefore, an *n*-degree polygon with interior angles 1 through to *n* is rigidly foldable if the following equation is satisfied:

2.9 $$\prod_{i=1}^{n}\mu_{i}=1.$$

This provides a method for determining if a polygon is rigidly foldable. First, fold-angle multipliers must be evaluated for each vertex. Then all fold-angle multipliers associated with a polygon must be multiplied to determine if equation (2.9) is satisfied. This method adds to the existing body of knowledge of determining rigid foldability [16–18,21,23,24] by providing a simple geometric interpretation that enables a straightforward approach to evaluating the rigid foldability of patterns made of degree-4 vertices. The value of the method is demonstrated here by evaluating the rigid foldability of existing tessellations and introducing new origami gadgets for creating new rigid foldable tessellations.

### 2.4 Rigidly foldable origami tessellations

An origami tessellation may contain many polygons. A tessellation can be rigidly foldable only if each of these polygons is rigidly foldable. However, an origami tessellation containing only rigidly foldable polygons may only be rigidly foldable over a restricted range because of global self-intersection (tessellations with inner portions cut out may not be rigidly foldable at all).

This method can be used to include additional information to crease patterns to demonstrate distinct fold angles in the tessellations. For each type of vertex, the fold angle multipliers ensure that certain pairs of fold angles are the same in the vertex, and we indicate sameness by adding a colour background to each fold line, choosing different colours for each distinct fold angle. Then it is clear to see that a given pattern is rigidly foldable by noting that the fold lines emanating from each vertex match in both fold direction and fold angle (magnitude) with the fold lines of each surrounding vertex. This not only demonstrates the rigid foldability of the tessellations, but is an example of the power of the fold angle multiplier method. One can both construct and prove rigid foldability by purely graphical techniques and inspection augmented by scalar algebra.

In this paper, we first evaluate existing flat-foldable tessellation to identify those that are rigidly foldable. We then present gadgets that facilitate the creation of rigidly foldable tessellations and show some resulting tessellations.

## 3. Known rigidly foldable tessellations

**Figure 2.**
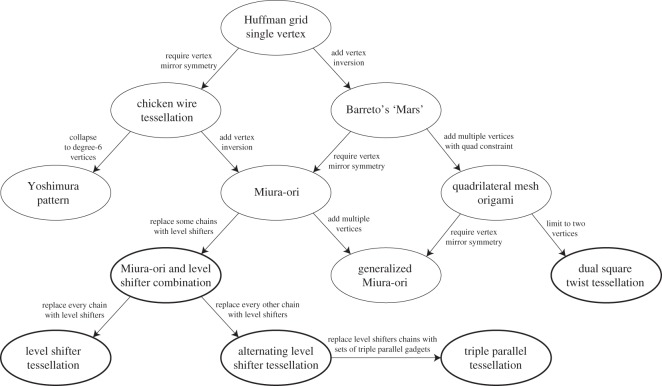
Rigidly foldable tessellations. New tessellations introduced in this paper are shown with a bold outline.

There are several origami tessellations which have been known to be rigidly foldable. Figure 2 shows an organization of rigidly foldable periodic tessellations. This chart does not represent a chronology of the development of these tessellations but rather a way to see how they are related. The goal of this diagram is not to present a strict tree-like taxonomy of organizing such patterns, but rather to draw together both known and new rigidly foldable patterns (these are introduced later in the paper) and illustrate some of the relationships among them. Although the intent of the figure is not to be a design guide for new tessellations, the relationships shown are examples of how existing tessellations can be modified to create new ones. These tessellations are described in the following sections.

### 3.1 Huffman grid

**Figure 3.**
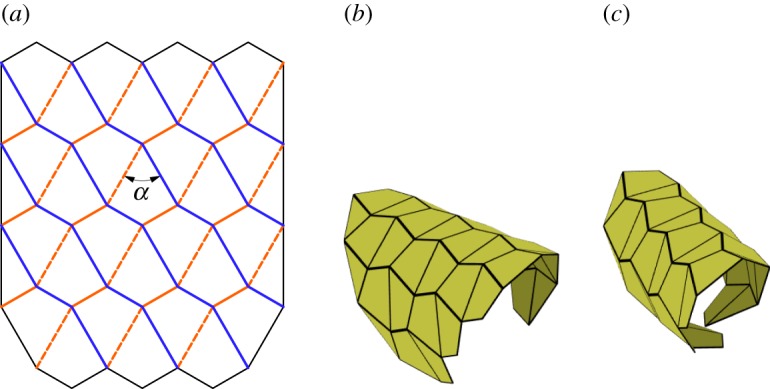
Huffman grid. Mountain folds are indicated by solid lines and valley folds by dashed lines. Fold lines of the same colour have the same fold angle. (*a*) Fold pattern, (*b*) partially folded position, and (*c*) pre-interference position.

The first tessellation we consider was presented by David Huffman [16], which we call the Huffman grid. This basic tessellation involves only a single degree-4 vertex that is rotated and repeated continuously through the tessellation as shown in figure 3*a*. Two of the sector angles are equal to *π*/2 and the other two are equal *α* and *π*−*α*. The tessellation is locally rigidly foldable for any *α*≠*π*/2. However, as seen in figure 3*c*, this tessellations folds into a cylindrical form and global self-intersection occurs before it reaches the final flat state.

Equations (2.5)–(2.8) show that opposite dihedral angles in a degree-4 vertex are equal in magnitude. This characteristic is useful in determining how many unique dihedral angles a tessellation contains. In this paper, we define all fold angles with equal magnitude to share one unique dihedral angle. At any position during deployment, this pattern contains fold angles of only two magnitudes; all fold lines with a positive slope in figure 3*a* have equal fold angle magnitude, as do all crease lines with a negative slope. As a result, this tessellation contains two unique dihedral angles.

**Figure 4.**
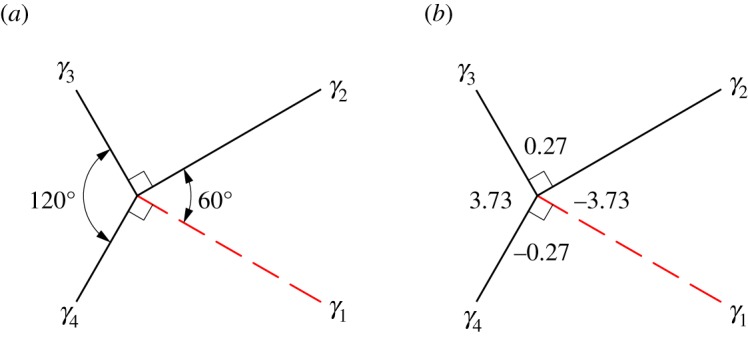
Single vertex of the Huffman grid. Solid lines indicate mountain folds and dashed lines indicate valley folds. (*a*) Sector angles and (*b*) fold-angle multipliers associated with the sectors.

We will use this tessellation to provide an example for the calculation of fold-angle multipliers and the evaluation of rigidly foldable polygons. Figure 4 shows a single vertex in the Huffman grid. Figure 4*a* shows the sector angles of this vertex and labels the dihedral angles *γ*_1_ through to *γ*_4_. The fold-angle multipliers associated with this vertex may be calculated using equations (2.5)–(2.8). For example, using equation (2.5)

3.1 $$\mu_{1}=\frac{\sin((1/2)(\pi /3+\pi /2))}{\sin((1/2)(\pi /3-\pi /2))}=-3.73.$$

The other fold-angle multipliers are then calculated using equations (2.6)–(2.8). The results are shown in figure 4*b*. For any polygon, this procedure is repeated for all vertices of the polygon. Because the Huffman grid includes only one unique vertex, further calculations are not necessary.

**Figure 5.**
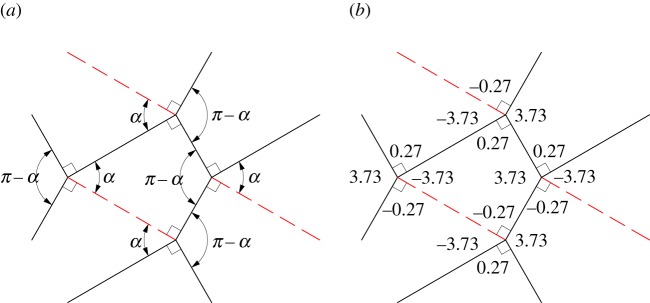
Single polygon of the Huffman grid. Solid lines indicate mountain folds and dashed lines indicate valley folds. (*a*) Sector angles and (*b*) fold-angle multipliers associated with the sectors. This polygon is rigidly foldable because (3.73)×(−0.27)×(−3.73)×(0.27)=1.

Figure 5 shows a single polygon of the tessellation with its associated fold-angle multipliers. This polygon is rigidly foldable because the product of all multipliers in the polygon is equal to 1. For any tessellation, this process is repeated for all unique enclosed polygons. Because the Huffman grid contains only one unique polygon, further calculations are not necessary and we conclude that it is locally rigidly foldable. Note that global rigid foldability is not ensured because of possible self-intersection.

### 3.2 Chicken wire tessellation

**Figure 6.**
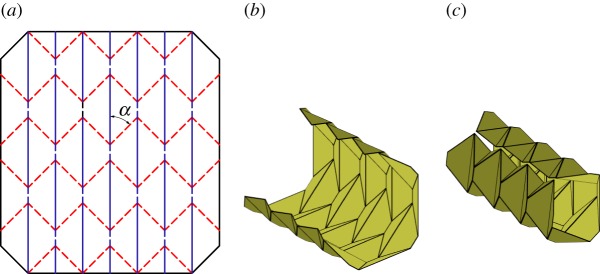
Chicken wire tessellation. Mountain folds are indicated by solid lines and valley folds by dashed lines. Fold lines of the same colour have the same fold angle. (*a*) Fold pattern, (*b*) partially folded position, and (*c*) pre-interference position.

The chicken wire tessellation (also known as the hexagonal pattern [26]) is constructed using a single vertex with mirror symmetry, as shown in figure 6. This tessellation is rigidly foldable for *α*<*π*/2 although it folds into a cylindrical form and global self-intersection occurs before a second flat position is obtained, as is seen in figure 6*c*. This tessellation contains two unique dihedral angles; all valley creases have the same fold angle, as do all mountain creases.

### 3.3 Barreto's ‘Mars’

**Figure 7.**
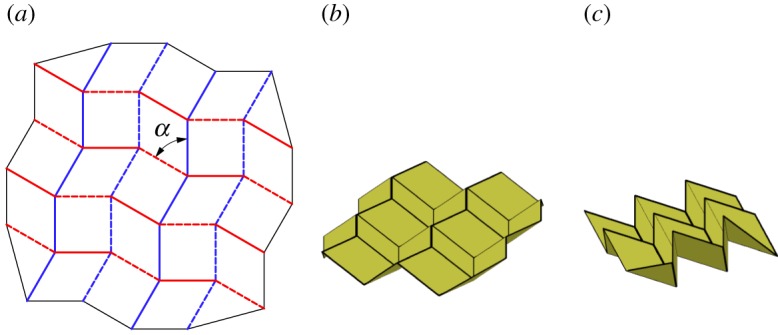
Barreto's ‘Mars’. Mountain folds are indicated by solid lines and valley folds by dashed lines. Fold lines of the same colour have the same fold angle. (*a*) Fold pattern, (*b*) partially folded position, and (*c*) mostly folded position.

Paulo Barreto presented a tessellation, ‘Mars’, which included a single degree-4 vertex and its inversion [27] as shown in figure 7. The vertex and its inversion are mirrored, rotated and repeated continuously through the tessellation. This tessellation includes square twists and parallelogram twists with configurations proved in [25] to be rigidly foldable. It is rigidly foldable for *α*<*π*/2. The pattern stays in a planar form, as shown in figure 7*b*,*c* and is able to reach its final flat state without self-intersection. This tessellation contains two unique dihedral angles; all vertical moving chains are equal in fold angle magnitude, as are all horizontal chains.

### 3.4 Miura-ori

**Figure 8.**
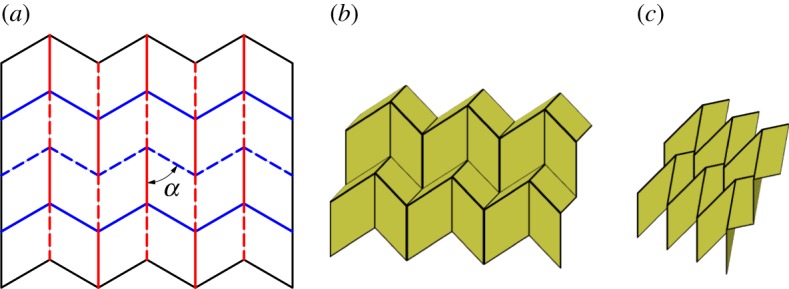
Miura-ori. Mountain folds are indicated by solid lines and valley folds by dashed lines. Fold lines of the same colour have the same fold angle. (*a*) Fold pattern, (*b*) partially folded position, and (*c*) mostly folded position.

The Miura-ori is an origami tessellation developed by Miura [1] which is comprised entirely from parallelograms. The tessellation is created by patterning a single degree-4 vertex and its inversion with a constraint of mirror symmetry of the vertex. As such, it is the result of a combination of the constraints on the chicken wire tessellation and Barreto's ‘Mars’ tessellation. This tessellation is rigidly foldable for any *α*<*π*/2. Figure 8*a* shows the fold pattern for the Miura-ori. The pattern stays in a planar form as shown in figure 8*b*,*c* and thus is able to reach its final flat state without self-intersection. The Miura-ori has two unique dihedral angles; all vertical creases are equal in angle, as are all other creases.

### 3.5 Yoshimura pattern

**Figure 9.**
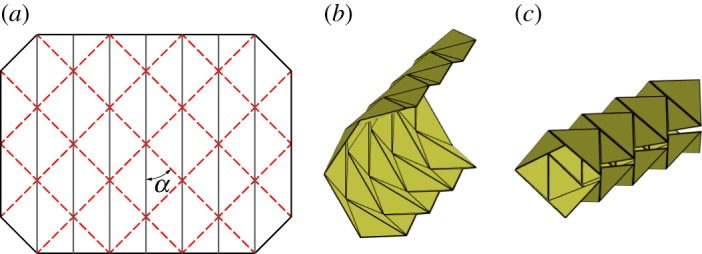
Yoshimura pattern. Mountain folds are indicated by solid lines and valley folds by dashed lines. Fold lines of the same colour have the same fold angle. (*a*) Fold pattern, (*b*) partially folded position, and (*c*) mostly folded position.

Yoshimura developed an origami pattern [28] based on the observed behaviour of thin cylinders under an axial buckling load. This pattern (also referred to as the diamond pattern [29]) is constructed by repeating a single degree-6 vertex with mirror symmetry. Unlike previously considered tessellations, this pattern has more than 1 d.f. due to the 3 d.f. in the degree-6 vertices. Figure 9*a* shows the fold lines for the Yoshimura pattern. Figure 9*b*,*c* shows one of the possible fold paths for this tessellation. Although the pattern is locally rigidly foldable for *α*<*π*/2, except under specific circumstances, global self-intersection occurs before the second flat state can be reached with this fold path.

As is noted in figure 2, the Yoshimura pattern may be obtained by collapsing the chicken wire tessellation into degree-6 vertices. Because this tessellation has more than 1 d.f., the number of unique dihedral angles is not constant. However, in the positions shown in figure 9, there are two unique fold angles; all valley creases have the same fold angle, as do all mountain creases.

### 3.6 Generalized quadrilateral mesh origami

**Figure 10.**
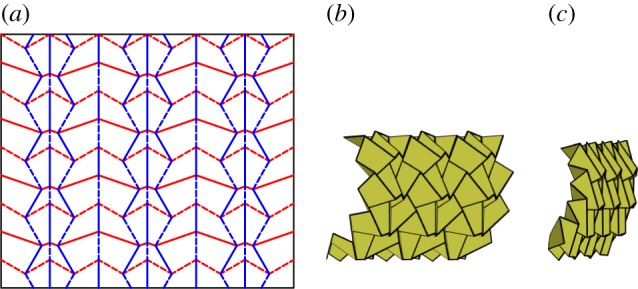
Quadrilateral meshed pattern. Mountain folds are indicated by solid lines and valley folds by dashed lines. Fold lines of the same colour have the same fold angle. (*a*) Fold pattern, (*b*) partially folded position, and (*c*) mostly folded position.

Tachi analysed quadrilateral mesh origami and presented conditions for rigid foldability [23]. As the name implies, this origami consists entirely of quadrilateral panels joined by creases meeting in degree-4 vertices. Quadrilateral mesh origami can also be evaluated using the method presented in [25], and may be rigidly foldable under the condition that equations (2.1) and (2.9) are satisfied for all vertices and polygons, respectively. Unlike the previously mentioned tessellations, this mesh does not need to include the repetition of a single, flat-foldable vertex, but may contain multiple vertices. Figure 10 shows one of the many possible rigidly foldable quadrilateral mesh patterns. Generalized quadrilateral mesh patterns may have any number of unique dihedral angles.

### 3.7 Generalized Miura-ori

**Figure 11.**
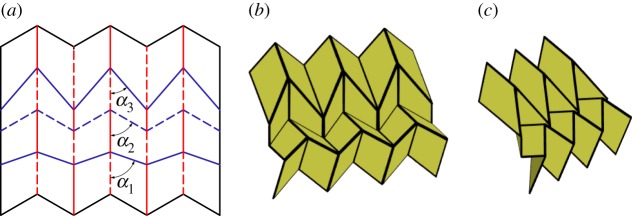
Generalized Miura-ori pattern. Mountain folds are indicated by solid lines and valley folds by dashed lines. Fold lines of the same colour have the same fold angle. (*a*) Fold pattern, (*b*) partially folded position, and (*c*) mostly folded position.

By requiring a quadrilateral mesh origami to contain vertices with mirror symmetry, the generalized Miura-ori is obtained (figure 11). The generalized Miura-ori differs from the Miura-ori in that it allows for multiple vertices. It can therefore be thought of as stemming from both the Miura-ori and the generalized quadrilateral mesh origami, as shown in figure 2. The generalized Miura-ori is globally rigidly foldable for all *α*_*i*_<*π*/2. The generalized Miura-ori may have many unique dihedral angles. All vertical creases have the same fold angle, while all chains of intersecting creases have another unique fold angle. (Chains with equal values of *α* have equal fold angles.)

## 4. Gadgets

Author Lang defined an origami *gadget* as a localized section of crease pattern that can replace an existing patch to add functionality or otherwise modify the pattern [30]. As a gadget replaces a single vertex or multiple vertices, it does not necessarily preserve the rest of the pattern, but may make manageable changes to surrounding creases. However, any rigidly foldable gadget which exactly replaces an existing rigidly foldable patch (all boundaries are equivalent) will not modify the motion of the pattern. Adding gadgets may also increase the complexity of the crease pattern by adding more folds.

The average polygon degree in a repeatable tessellation containing only degree-4 vertices must be equal to 4 (see appendix A). As a result, any tessellation constructed using only degree-4 vertices and featuring polygons of more than fourth degree must also contain triangles. Because of this, of particular interest are gadgets which include triangles and allow for the use of *n*>4 polygons in rigidly foldable tessellations.

### 4.1 Corner gadget

**Figure 12.**
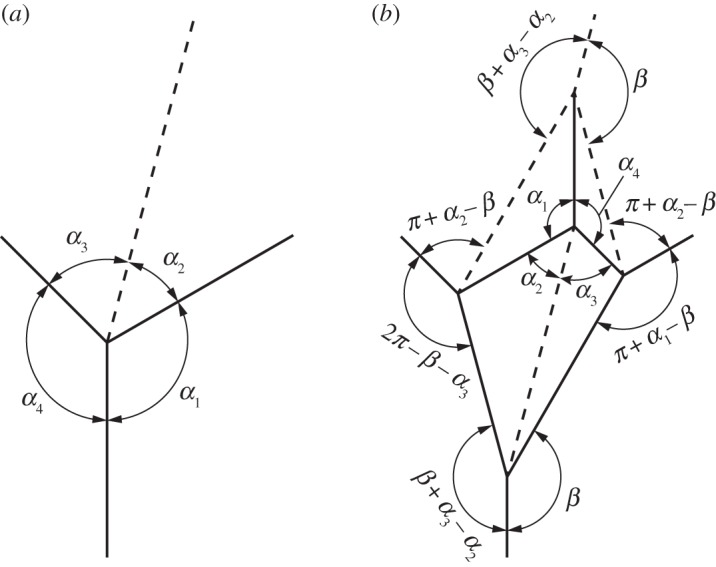
Corner gadget. This gadget is rigidly foldable for *π*/2<*α*_1_<*β*<*π*. The value of *β* does not have any effect on the fold angle multipliers between exterior creases. Solid lines indicate mountain folds and dashed lines indicate valley folds. (*a*) Original vertex and (*b*) gadget.

**Figure 13.**
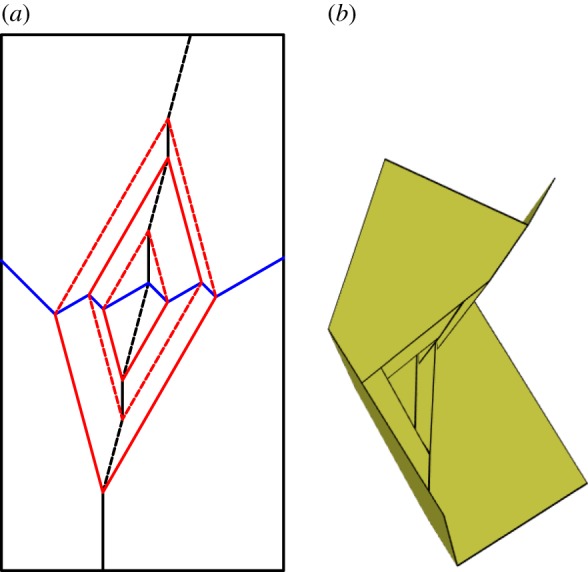
Recursive corner gadget. Note that each successive interior vertex is a rotation of the previous interior vertex by 180°. Mountain folds are indicated by solid lines and valley folds by dashed lines. Fold lines of the same colour have the same fold angle. (*a*) Fold pattern and (*b*) partially-folded state.

A flat-foldable degree-4 vertex may be modified to create a rigidly foldable network of four degree-4 vertices with four outwardly extending creases in the same location as the original creases as shown in figure 12. The fold angle multipliers between these four creases remain the same as in the original vertex, therefore, the overall motion remains the same. As such, this gadget follows the conventional definition of a gadget. This gadget is the result if a vertex is depressed into a second rigidly foldable form. The depression can be repeated as many times as is desired as shown in figure 13. The value of *β* changes the size and shape of the depression, but does not change the fold angle multipliers between any of the exterior creases. It is required that *β*>*α*_1_. Replacing a vertex with a corner gadget adds one unique dihedral angle to the tessellation. (The folds along the boundaries of the quadrilateral in the corner gadget are all of equal fold angle.)

#### 4.1.1 Symmetric corner gadget

**Figure 14.**
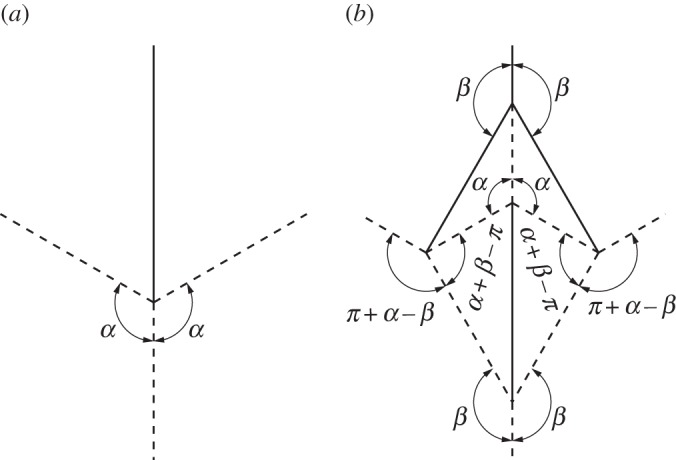
Symmetric corner gadget. This gadget is rigidly foldable for *π*/2<*α*<*β*< *π*. The value of *β* does not have any effect on the fold angle multipliers between exterior creases. Solid lines indicate mountain folds and dashed lines indicate valley folds. (*a*) Original vertex and (*b*) gadget.

A special case of the corner gadget is one where the minor crease lines of the original vertex are collinear. As a result, the gadget created is symmetric about this crease line as seen in figure 14. As with the general corner gadget, the fold angle multipliers between the exterior creases remain the same as in the original vertex, regardless of the value of *β*. As with the original, it is also required that *β*>*α*.

### 4.2 Triple parallel gadget

**Figure 15.**
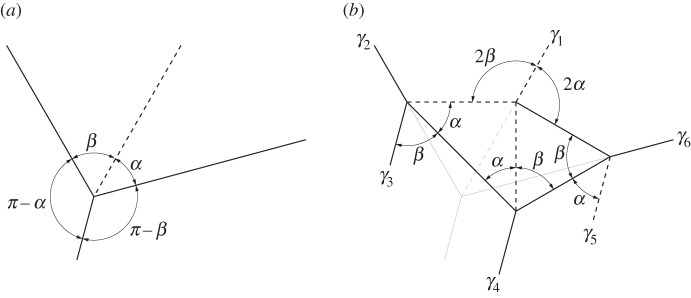
Triple parallel gadget. This gadget is rigidly foldable for 0<*α*,*β*<*π*/2 and *α*≠*β*. The original vertex is superimposed with light weight on the gadget in (*b*). Solid lines indicate mountain folds and dashed lines indicate valley folds. (*a*) Original vertex and (*b*) gadget.

Any flat-foldable degree-4 origami vertex which does not contain two collinear crease lines and does not contain 90° sector angles may be modified to become a network of four vertices enclosing two isosceles triangles. This network contains six outwardly extending creases, three of which are parallel to one of the original minor creases and the other three are each coincident to each of the other three original creases (figure 15). The fold angle multipliers between the three non-parallel creases remain the same as in the original vertex. The two non-adjacent parallel creases have dihedral angles equal in magnitude but opposite in sign. Replacing a vertex with a triple parallel gadget adds one unique dihedral angle. The middle parallel crease has a dihedral angle equal to the original crease, while the outer parallel creases are equal in magnitude to each other.

Labelling the opposite crease as crease number one and numbering counter-clockwise (figure 15), the multipliers are as follows:

4.1 $$\begin{array}{rl} \mu_{1} & \equiv \frac{\tan((1/2)\gamma_{2})}{\tan((1/2)\gamma_{1})}=-\frac{\cos((1/2)(\alpha -\beta ))}{\cos((1/2)(\alpha +\beta ))}, \end{array}$$

4.2 $$\begin{array}{rl} \mu_{2} & \equiv \frac{\tan((1/2)\gamma_{3})}{\tan((1/2)\gamma_{2})}=-\frac{\sin((1/2)(\alpha -\beta ))}{\sin((1/2)(\alpha +\beta ))}, \end{array}$$

4.3 $$\begin{array}{rl} \mu_{3} & \equiv \frac{\tan((1/2)\gamma_{4})}{\tan((1/2)\gamma_{3})}=-\frac{\sin(\alpha +\beta )}{\sin(\alpha -\beta )}, \end{array}$$

4.4 $$\begin{array}{rl} \mu_{4} & \equiv \frac{\tan((1/2)\gamma_{5})}{\tan((1/2)\gamma_{4})}=\frac{\sin(\alpha -\beta )}{\sin(\alpha +\beta )}, \end{array}$$

4.5 $$\begin{array}{rl} \mu_{5} & \equiv \frac{\tan((1/2)\gamma_{6})}{\tan((1/2)\gamma_{5})}=\frac{\sin((1/2)(\alpha +\beta ))}{\sin((1/2)(\alpha -\beta ))} \end{array}$$

4.6 $$\begin{array}{rl} \;\text{and}\;\;\mu_{6} & \equiv \frac{\tan((1/2)\gamma_{1})}{\tan((1/2)\gamma_{6})}=-\frac{\cos((1/2)(\alpha +\beta ))}{\cos((1/2)(\alpha -\beta ))}. \end{array}$$

**Figure 16.**
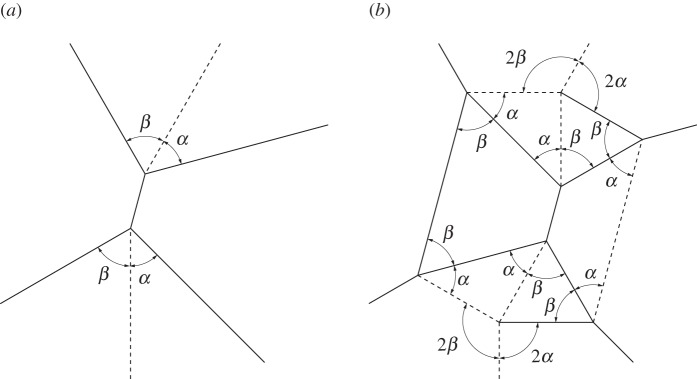
A set of triple parallel gadgets. Solid lines indicate mountain folds and dashed lines indicate valley folds. (*a*) Original vertices and (*b*) set of gadgets.

Of particular interest is an arrangement where two of these gadgets are combined in such a way that the three parallel crease lines from one gadget meet the three parallel crease lines from another. We call this a set of triple parallel gadgets, shown in figure 16*b*. To form a set, the two gadgets must have equal values for *α* and *β*. A set of triple parallel gadgets may replace a combination of two vertices which are mirror-symmetric, as shown in figure 16. A set of these gadgets adds a third dihedral angle to the set of vertices (figure 16*a*) where this third angle is entirely contained in the set of gadgets.

### 4.3 Level shifters

**Figure 17.**
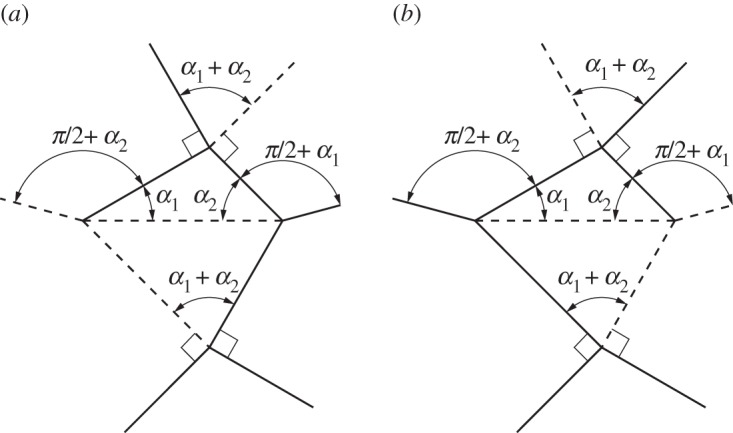
Asymmetric level shifter. This gadget is rigidly foldable for 0<*α*_1_,*α*_2_<*π*/2 and *α*_1_+*α*_2_≠*π*/2. (*a*,*b*) Two possible crease assignments. The gadget may also be mirrored across itself and remains rigidly foldable. All vertices in this gadget are flat foldable (opposite sector angles sum to *π*). Solid lines indicate mountain folds and dashed lines indicate valley folds. (*a*) Crease type 1 and (*b*) crease type 2.

A level shifter is an origami gadget that has found use in formal origami design and shows up often in origami in the form of a ‘spread sink’ [30]. As its name suggests it is used to bring together two sections of an origami pattern which are at different levels. Level shifters allow for selective widening of origami patterns [30]. A level shifter in its most basic form is rigidly foldable. Figure 17 shows an asymmetric level shifter. Two independent input angles (*α*_1_,*α*_2_) fully define the gadget. The gadget is flat foldable, therefore, all other angles may be calculated by recalling that opposite sector angles in a vertex sum to *π*. A chain of level shifters has three unique fold angles. Two angles intertwine along the direction of the chain and a third angle passes through the horizontal creases.

#### 4.3.1 Symmetric level shifter

**Figure 18.**
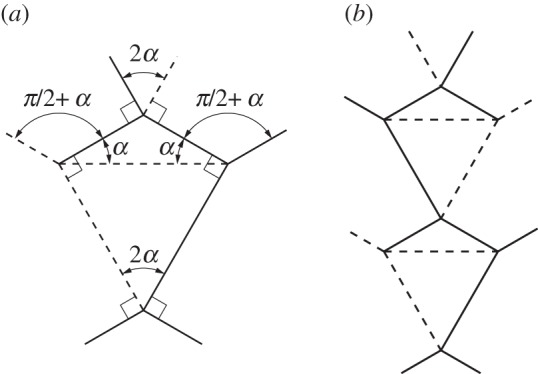
Symmetric level shifter. This gadget is rigidly foldable for 0<*α*<*π*/2 and *α*≠*π*/4. This gadget may be repeated by mirroring itself and repeating as seen in (*b*). Solid lines indicate mountain folds and dashed lines indicate valley folds. (*a*) Level shifter and (*b*) level shifter repeatability.

A special case of the level shifter gadget occurs when *α*_1_=*α*_2_. This creates a symmetric level shifter and we use *α* to designate the input angle. The dihedral angles for each of the horizontal crease lines in figure 18*b* are equal. Therefore, this gadget may be useful in creating large tessellations which do not self-intersect.

## 5. Directly modified rigidly foldable patterns

In this section, we explore modifications that can be made to existing rigidly foldable tessellations. These modifications use the gadgets presented previously to directly replace existing portions of the tessellations.

### 5.1 Miura-ori with corner gadget

**Figure 19.**
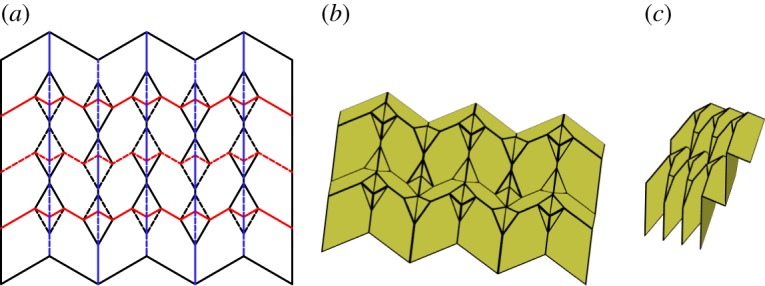
Miura-ori pattern with corner gadgets. Mountain folds are indicated by solid lines and valley folds by dashed lines. Fold lines of the same colour have the same fold angle. (*a*) Fold pattern, (*b*) partially folded position, and (*c*) mostly folded position.

By replacing the vertices of a Miura-ori tessellation with corner gadgets, the tessellation shown in figure 19 is created. This tessellation has the same overall motion as the Miura-ori; however, the dimensions of the tessellation in its final, folded state are changed, as can be seen by comparing figures 8*c* and 19*c*. Also, when constructed using hinges with finite stiffness, this configuration is stiffer than the original Miura-ori because of the addition of springs in parallel.

### 5.2 Baretto's ‘Mars’ with corner gadget

**Figure 20.**
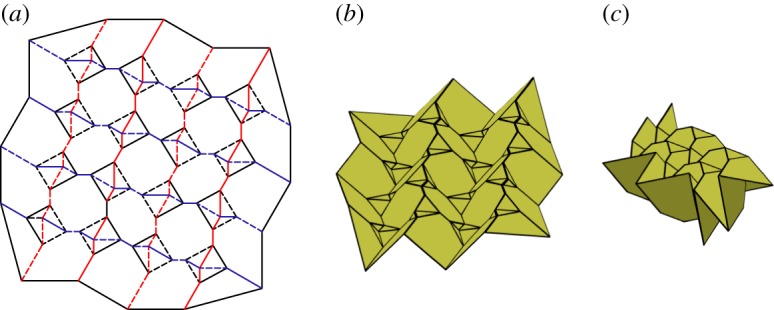
Barreto's ‘Mars’ with corner gadgets. Mountain folds are indicated by solid lines and valley folds by dashed lines. Fold lines of the same colour have the same fold angle. (*a*) Fold pattern, (*b*) partially folded position, and (*c*) mostly folded position.

As with the Miura-ori, the ‘Mars’ tessellation may be modified by replacing vertices with corner gadgets. Such a resulting tessellation is shown in figure 20. This tessellation has the same motion as the ‘Mars’, however, like with the modified Miura-ori, the footprint of the final flat position is changed.

## 6. New rigidly foldable patterns

We present several new rigidly foldable patterns in this section. Many of these patterns were constructed using the previously mentioned gadgets.

### 6.1 Dual square twist tessellation

**Figure 21.**
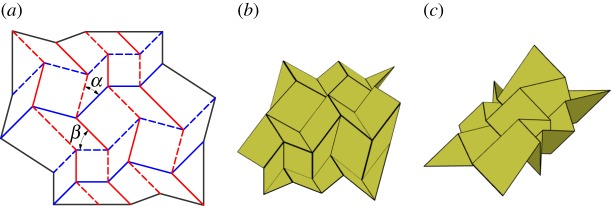
Dual square twist tessellation. Mountain folds are indicated by solid lines and valley folds by dashed lines. Fold lines of the same colour have the same fold angle. (*a*) Fold pattern, (*b*) partially folded position, and (*c*) mostly folded position.

A special case of a generalized quadrilateral mesh origami occurs when only two different vertices are used. These vertices, along with their inversions are used to create the tessellation shown in figure 21. This tessellation contains many repetitions of square twists with two twist angles. It is rigidly foldable for 0<*α*≤*β*<90°. Note that the case where *α*=*β* results in the ‘Mars’ tessellation. This tessellation contains four unique dihedral angles. Every other set of mostly vertical chains have equal fold angle magnitudes, as do every other set of mostly horizontal chains.

### 6.2 Alternating level shifter tessellation

**Figure 22.**
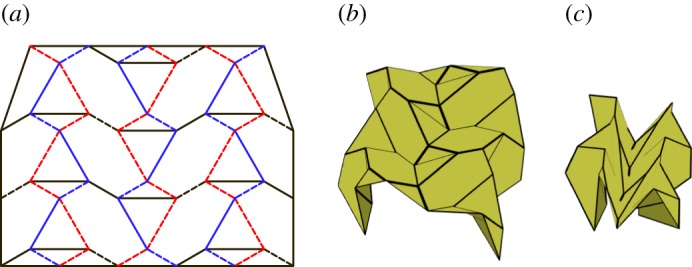
Alternating level shifter tessellation. Mountain folds are indicated by solid lines and valley folds by dashed lines. Fold lines of the same colour have the same fold angle. (*a*) Fold pattern, (*b*) partially folded position, and (*c*) mostly folded position.

**Figure 23.**
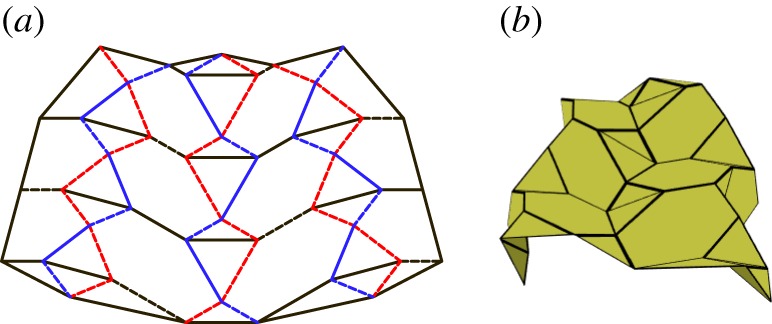
Non-parallel alternating level shifter tessellation. Mountain folds are indicated by solid lines and valley folds by dashed lines. Fold lines of the same colour have the same fold angle. (*a*) Fold pattern and (*b*) partially folded position.

This tessellation is created by attaching chains of level shifters in opposite directions. The chains are arranged so that adjacent chains are in alternate directions and are connected using hexagons (figure 22). This tessellation is rigidly foldable for any rigidly foldable level shifter. Figure 22 shows the simplest form of this tessellation, however, chains of different level shifters may also be joined together. As is shown in figure 23, these chains will not be parallel. This tessellation contains two unique fold angles for each uniquely angled level shifter chain and another unique fold angle for all of the creases between chains.

### 6.3 Level shifter tessellation

**Figure 24.**
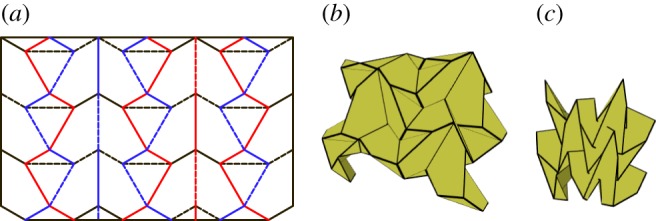
Level shifter tessellation. Mountain folds are indicated by solid lines and valley folds by dashed lines. Fold lines of the same colour have the same fold angle. (*a*) Fold pattern, (*b*) partially folded position, and (*c*) mostly folded position.

**Figure 25.**
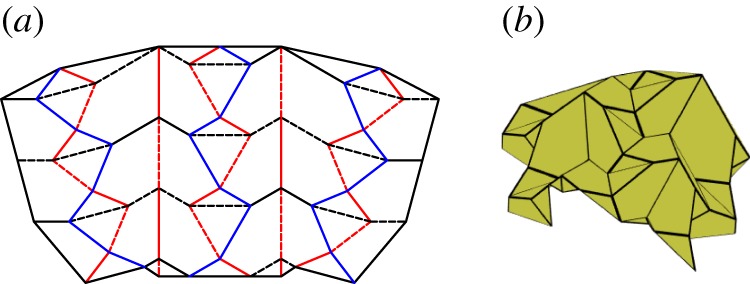
Non-parallel level shifter tessellation. Mountain folds are indicated by solid lines and valley folds by dashed lines. Fold lines of the same colour have the same fold angle. (*a*) Fold pattern and (*b*) partially folded position.

This tessellation is created by attaching chains of level shifters in the same direction. These chains are attached using pentagons (figure 24). This tessellation is rigidly foldable for any rigidly foldable level shifter. As with the previously mentioned tessellation, this tessellation may also include chains of level shifters with different angles and still be rigidly foldable. However, in this case, each chain remains parallel, while the crease lines separating the chains are no longer parallel (figure 25). This tessellation contains two unique fold angles for each uniquely angled level shifter chain, and another unique fold angle for all of the creases between chains. Also, each set of collinear folds may have its own unique dihedral angle or it may be equal to another set, depending on the orientation.

### 6.4 Miura-ori and level shifter combination

**Figure 26.**
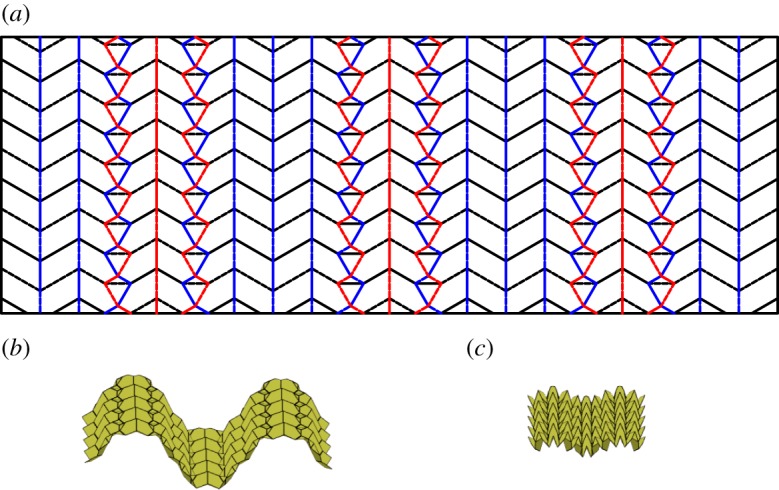
Combination of Miura-ori and level shifter patterns. Mountain folds are indicated by solid lines and valley folds by dashed lines. Fold lines of the same colour have the same fold angle. (*a*) Fold pattern, (*b*) partially folded position, and (*c*) mostly folded position.

Level shifter chains may be combined with Miura-ori patterns to construct new tessellations such as that shown in figure 26. When this occurs, level shifter chains with mountain folds separating the triangles act as valley-like folds during the intermediate folding positions as shown in the centre in figure 26*b*. Likewise, level shifter chains with valley folds separating the triangles act as mountain-like folds during the intermediate positions as shown on the right and left in figure 26*b*. As the pattern approaches the final position, these level shifter chains become flat again as shown in figure 26*c*. The tessellation shown has four unique fold angles (two that intertwine on each level shifter chain, one for each collinear chain and a third for all connecting creases).

### 6.5 Triple parallel tessellation

**Figure 27.**
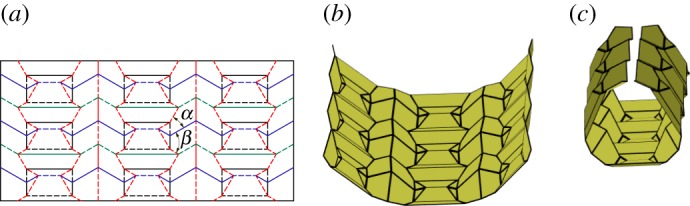
Triple parallel tessellation. Mountain folds are indicated by solid lines and valley folds by dashed lines. Fold lines of the same colour have the same fold angle. (*a*) Fold pattern, (*b*) partially folded position, and (*c*) mostly folded position.

As with the level shifter gadget, the triple parallel gadget may also be used in many different combinations with the Miura-ori pattern. Figure 27*a* provides one example where alternating chains of the Miura-ori are replaced with chains of back-to-back level shifters. The gadgets are arranged so that the three parallel folds from each gadget meet the three parallel folds from another gadget. This tessellation is rigidly foldable under the same conditions that the gadgets are rigidly foldable. However, as is shown in figure 27*c*, global self-intersection occurs before the final flat state. This tessellation includes three unique fold angles; the vertical crease chains are of equal angle, all folds stemming from these chains are of equal angle, and each set of triple parallel gadgets forms a third fold angle. (This third angle follows the rectangles formed by the set of triple parallel gadgets.)

## 7. Conclusion

This paper has identified and categorized existing flat-foldable rigidly foldable origami tessellations. Using the method of fold-angle multipliers, several origami *gadgets* have been designed which may facilitate the modification of creation of rigidly foldable origami tessellations. New flat-foldable and rigidly foldable origami tessellations involving these gadgets have been presented. These tessellations have final geometries that are more compact in one dimension than their counterparts. Because rigid foldability can be a critical feature for origami applications in materials other than paper, the identification of rigidly foldable tessellations, introduction of rigidly foldable gadgets and examples of how to create new tessellations by combining these concepts lays the foundation for future application.

## Figures and Tables

**Figure 28.**
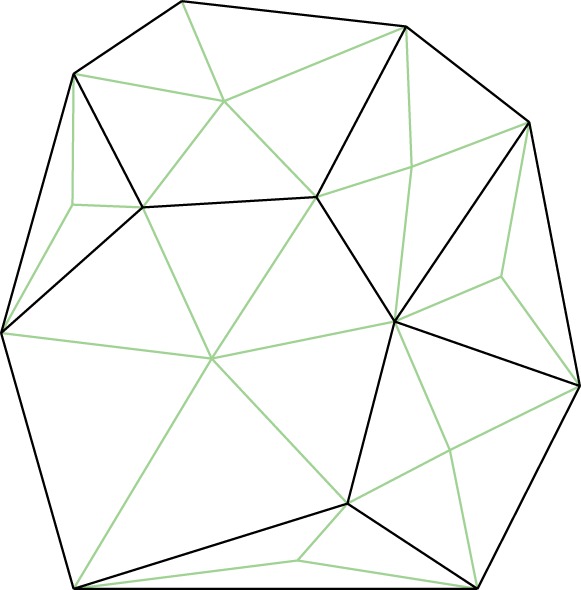
Arbitrary polygon mesh with subdividing triangles.

## Data accessibility

The results of the article are reproducible from the data reported in the manuscript and the appendix.

## Appendix A. Proof of average polygon size

We will consider a mesh of *k* polygons with *n*_*i*_ as the number of vertices of the *i*th polygon. (Such a mesh is shown in black in figure 28.) Now we divide each polygon into *n*_*i*_ triangles which have a common vertex at the centre of the polygon (shown in green). Let *V* , *E* and *F* be the number of vertices, edges and polygons of the original graph (black), respectively. Let *d* be the degree of all interior vertices.

If we sum over all polygons, we can calculate the number of triangles *N* as follows:

A 1 $$N=\sum_{i=1}^{k}n_{i}.$$

Second, we sum over the edges. Each interior edge contributes two triangles, while each boundary edge contributes only one. If there are *E*_b_ edges on the boundary, then

A 2 $$N=2E-E_{b}.$$

Solving for *E* yields

A 3 $$E=\frac{1}{2}(N+E_{b}).$$

Third, we sum over vertices. Assume that all interior vertices have degree *d* and boundary vertices have degree *d*_*j*_ (where *j* is the index of a boundary vertex). If *m* is the number of boundary vertices, then

A 4 $$N=dV-\sum_{j=1}^{m}(d-d_{j}).$$

Solving for *V* results in

A 5 $$V=\frac{1}{d}\left(N+\sum_{j=1}^{m}(d-d_{j})\right).$$

According to the Euler relation,

A 6 V−E+F=2.

Solving for *F* yields

A 7 F=2+E−V.

Substituting in equations (A 3) and (A 5) results in

A 8 $$F=2+\frac{1}{2}(N+E_{b})-\frac{1}{d}\left(N+\sum_{j=1}^{m}(d-d_{j})\right).$$

Rearranging,

A 9 $$F=N\left(\frac{1}{2}-\frac{1}{d}\right)+\frac{1}{2}E_{b}-\frac{1}{d}\sum_{j=1}^{m}(d-d_{j})+2.$$

Now the average polygon size *n*_avg_ may be found:

A 10 $$n_{\mathrm{avg}}=\frac{\sum_{i=1}^{F}n_{i}}{\sum_{i=1}^{F}1}=\frac{N}{F}.$$

Substituting in equation (A 8) yields

A 11 $$n_{\mathrm{avg}}=\frac{N}{N(1/2-1/d)+(1/2)E_{b}-(1/d)\sum_{j=1}^{m}(d-d_{j})+2}.$$

Dividing both numerator and denominator by *N* results in

A 12 $$n_{\mathrm{avg}}=\frac{1}{\left((d-2)/2d)+(1/N)((1/2)E_{b}-(1/d)\sum_{j=1}^{m}(d-d_{j})+2\right)}.$$

As the number of triangles (*N*) approaches infinity, we reach the following:

A 13 $$\lim_{N\to \infty }n_{\mathrm{avg}}=\frac{2d}{d-2}.$$

Thus for *d*=4, *n*_avg_→4. Note also that for *d*=3, *n*_avg_→6 and for *d*=6, *n*_avg_→3 as is found in the diamond tessellation. This equation is similar to the equation for the value of the interior angles of a regular polygon. As a result, *n*_avg_ is equal to the degree of the regular polygon that has interior angles equal to 2*π*/*d*. For example, an array of regular hexagons of equal size contains only degree-3 vertices. Note that these conditions only hold as the number of polygons approach infinity. Therefore, degree-4 tessellations can be created which do not have an average polygon size of four if these tessellations do not repeat continuously.
